# Hypospadias and maternal exposure to atrazine via drinking water in the National Birth Defects Prevention study

**DOI:** 10.1186/s12940-016-0161-9

**Published:** 2016-07-15

**Authors:** Jennifer J. Winston, Michael Emch, Robert E. Meyer, Peter Langlois, Peter Weyer, Bridget Mosley, Andrew F. Olshan, Lawrence E. Band, Thomas J. Luben, the National Birth Defects Prevention Study

**Affiliations:** Carolina Population Center, University of North Carolina at Chapel Hill, Chapel Hill, NC USA; Department of Geography and Carolina Population Center, University of North Carolina at Chapel Hill, Chapel Hill, NC USA; North Carolina Birth Defects Monitoring Program, State Center for Health Statistics, Raleigh, NC USA; Department of Maternal and Child Health, University of North Carolina at Chapel Hill, Chapel Hill, NC USA; Texas Department of State Health Services, Birth Defects Epidemiology and Surveillance Branch, Austin, TX USA; Center for Health Effects of Environmental Contamination, University of Iowa, Iowa City, IA USA; Department of Pediatrics, Arkansas Children’s Hospital, Little Rock, AR USA; Department of Epidemiology, University of North Carolina at Chapel Hill, Chapel Hill, NC USA; Department of Geography and Institute for the Environment, University of North Carolina at Chapel Hill, Chapel Hill, NC USA; National Center for Environmental Assessment, United States Environmental Protection Agency, Research Triangle Park, NC USA

**Keywords:** Hypospadias, Birth defects, Atrazine, Groundwater, Surface water, Endocrine disruptors

## Abstract

**Background:**

Hypospadias is a relatively common birth defect affecting the male urinary tract. It has been suggested that exposure to endocrine disrupting chemicals might increase the risk of hypospadias by interrupting normal urethral development.

**Methods:**

Using data from the National Birth Defects Prevention Study, a population-based case-control study, we considered the role of maternal exposure to atrazine, a widely used herbicide and potential endocrine disruptor, via drinking water in the etiology of 2nd and 3rd degree hypospadias. We used data on 343 hypospadias cases and 1,422 male controls in North Carolina, Arkansas, Iowa, and Texas from 1998–2005. Using catchment level stream and groundwater contaminant models from the US Geological Survey, we estimated atrazine concentrations in public water supplies and in private wells. We assigned case and control mothers to public water supplies based on geocoded maternal address during the critical window of exposure for hypospadias (i.e., gestational weeks 6–16). Using maternal questionnaire data about water consumption and drinking water, we estimated a surrogate for total maternal consumption of atrazine via drinking water. We then included additional maternal covariates, including age, race/ethnicity, parity, and plurality, in logistic regression analyses to consider an association between atrazine and hypospadias.

**Results:**

When controlling for maternal characteristics, any association between hypospadias and daily maternal atrazine exposure during the critical window of genitourinary development was found to be weak or null (odds ratio for atrazine in drinking water = 1. 00, 95 % CI = 0.97 to 1.03 per 0.04 μg/day increase; odds ratio for maternal consumption *=* 1.02, 95 % CI = 0.99 to 1.05; per 0.05 μg/day increase).

**Conclusions:**

While the association that we observed was weak, our results suggest that additional research into a possible association between atrazine and hypospadias occurrence, using a more sensitive exposure metric, would be useful.

**Electronic supplementary material:**

The online version of this article (doi:10.1186/s12940-016-0161-9) contains supplementary material, which is available to authorized users.

## Background

Hypospadias is a relatively common birth defect of the male urinary tract, affecting between 4 to 6 of every 1,000 male births [1]. It occurs as a result of abnormal urethral closure during gestational weeks 8–14, and manifests with a urethral opening on the underside of the penis [1]. It has a significant personal and public health impact, as surgical repair is often needed to allow for normal urinary and sexual function, and even after correction, hypospadias may result in psychosocial and sexual problems later in life [2].

Normal urethral closure during fetal development depends upon binding of testosterone to the androgen receptor and subsequent action by the androgen receptor [1]. It has therefore been suggested that endocrine disrupting chemicals might increase hypospadias risk [1]. One potential endocrine disrupting chemical that has been examined for an association with genitourinary malformations is atrazine, one of the most widely used agricultural herbicides in the United States [3]. It has been suggested that atrazine may affect aromatase levels, and by extension alter testosterone metabolism and sexual differentiation in frogs [4, 5], and there is experimental evidence to support a link between atrazine and genitourinary malformations in both rats [6] and amphibians [4, 5, 7, 8]. These effects in animals may be analogous to some of the key events that could lead to hypospadias in a developing fetus, providing biological plausibility for an association between exposure to atrazine and hypospadias. The evidence to document a specific link between hypospadias and atrazine in humans is somewhat equivocal, however. Winchester et al. found an elevated prevalence of “other urogenital anomalies,” but not of “malformed genitalia” among infants conceived during months of the highest concentrations of atrazine and other chemicals measured by the US Geological Survey’s National Water Quality Assessment Program [9]. Chevrier et al. examined urinary biomarkers of atrazine and general male genital anomalies. They found an increase in male genital anomalies among mothers with quantifiable atrazine or atrazine metabolites in urine, but their sample size was very small (5 cases exposed and 18 case unexposed) [10]. Only two published studies to date have looked specifically at atrazine and hypospadias in humans, and they found mixed results. The first study, by Meyer et al., assigned maternal exposure to several agricultural pesticides (including atrazine) by estimating the amount of pesticides applied within a 500–meter buffer of the mother’s home. They did not find evidence of an association between hypospadias and atrazine [11]. The second study, by Agopian et al., used Texas birth defects registry data and assigned atrazine levels to mothers based on their county of residence at birth. They found some evidence of an increased risk of second or third degree hypospadias for mothers in the 25th–75th percentiles of exposure (odds ratio = 1.52; CI = 1.25–1.85) and for the 75th–90th percentiles of exposure (odds ratio = 1.44; CI = 1.11–1.85), but suggested that further research was needed to confirm the mechanism for an association between hypospadias and county level atrazine use [12].

In this study, we seek to build on existing research examining the potential relationship between atrazine and hypospadias by incorporating information about maternal water consumption, as well as other known demographic and behavioral risk factors. We use a novel technique to estimate maternal exposure to atrazine in drinking water, and take advantage of unique data that include information about behavioral covariates to control for confounding and maternal residential address throughout pregnancy to improve exposure assessment.

## Methods

### Study population

Data from this study come from the National Birth Defects Prevention Study (NBDPS), a population-based case-control study conducted in ten states with the Centers for Disease Control and Prevention. NBDPS identifies second- and third-degree hypospadias cases, which are considered moderate to severe [1], from birth defect surveillance registries and randomly selects controls from birth certificates or birth hospital records. NBDPS does not include first-degree, or mild, hypospadias cases due to variable diagnosis patterns and medical documentation. NBDPS cases were contributed by each center (an active surveillance birth defects registry), and reviewed by a clinical geneticist there to ensure that all cases met NBDPS study criteria and thus ensure accurate and consistent case ascertainment across study sites. Second- and third- degree cases of hypospadias from all centers were then reviewed in detail by a clinical geneticist who focused on this birth defect, and who considered medical record information and anatomical descriptions provided by health care providers [13].

NBDPS also collects data on a wide number of covariates via computer-assisted telephone interview with the mother. For the years included in the study, the interview also included a water module which asked questions about drinking water source and water consumption. Additional covariates include maternal address throughout pregnancy, and a number of known risk factors for hypospadias, including maternal age, maternal race/ethnicity, parity, plurality, maternal choline intake, and use of fertility medications [14].

The study included hypospadias cases (*n =* 343, of which 305 were isolated cases where hypospadias was the only observed anomaly, and 38 were cases where other birth defects were observed in conjunction with hypospadias) and male controls (*n =* 1,422) whose mothers were successfully interviewed from North Carolina, Iowa, Arkansas, and Texas. Iowa, Arkansas, and Texas contributed data for women with estimated due dates between 1998 and 2005. North Carolina did not join the study until 2003, providing data for women with estimated due dates between 2003 and 2005. Analyses were conducted on isolated and non-isolated cases combined. Among cases identified in the clinical database, participation rates in the full interview were 73 % for Arkansas, 44 % for Iowa, 73 % for North Carolina, and 65 % for Texas. Participation rates for the male and female control groups were 67 % for Arkansas, 63 % for Iowa, 72 % for North Carolina, and 64 % for Texas.

The 4 states were selected from the NBDPS study sites because any association between atrazine and hypospadias was predicted to be small, and atrazine concentrations in streams were predicted to be higher in Iowa, Arkansas, Texas, and North Carolina than in other study sites [15]. The time period was selected because data were collected for water consumption during this time.

### Atrazine exposure estimation

We estimated atrazine concentrations using two United States Geological Survey (USGS) models [3, 16]. Because many public water supplies treat their raw water with various filtration processes that will reduce concentrations of atrazine, these models overestimate atrazine in drinking water. We selected this approach, however, because the models allowed us to consider a full range of atrazine concentrations, and monitoring data were not available for concentrations falling below the US Environmental Protection Agency’s maximum contaminant level of 3 μg/L.

We assigned atrazine concentrations to public water supplies based on the type and location of the water intakes for each utility. Geographic coordinates of surface and groundwater intakes for public water utilities were available for Iowa, Texas, and North Carolina [17–20]. For Arkansas, we used Google Earth to geocode water intakes using descriptions of intake locations available from the Arkansas Department of Health [21]. For water supplies using surface water, we used estimated annual mean atrazine concentrations in streams predicted by the Watershed Regressions for Pesticides (WARP) model [16]. WARP uses estimated watershed-level atrazine use, the percentage of the watershed’s agricultural land with a soil restrictive layer near the surface, total precipitation during May and June of the sampling year, rainfall erosivity for the watershed, and streamflow caused by precipitation on saturated soil in order to generate nationwide estimates of atrazine concentrations in streams. We used WARP estimates from the nearest stream reach to assign an annual mean atrazine concentration to public water intakes.

For water supplies using groundwater, we used site-variable model predictions from the “Regression Models for Estimating Concentrations of Atrazine plus Deethylatrazine in Shallow Groundwater in Agricultural Areas of the United States” [3]. This model uses groundwater residence time, atrazine use intensity, artificial drainage practices, depth to the seasonally high water-table, content of the uppermost soil content, soil permeability, groundwater recharge rates, and well depth to provide gridded estimates of atrazine concentrations in shallow groundwater. For groundwater intakes, and for mothers in NBDPS using private wells, we used gridded atrazine predictions from the USGS groundwater model and bilinear interpolation to estimate atrazine concentrations based on the grid cell where the intake was located and the adjacent grid cells.

Each public water utility was then assigned an atrazine concentration equal to the mean of the predicted atrazine concentrations for all of the intakes for that utility. Geographic assignment was conducted using ArcGIS version 10.2 (ESRI Inc., Redlands, CA, USA).

### Exposure assignment to study participants

We based our exposure assessment on maternal residential addresses during weeks 6–16 postconception, which encompass the critical period for urethral development [22]. Mothers using a public water supply were assigned a water utility for each reported residential address by the University of Iowa Center for Health Effects of Environmental Contamination (CHEEC), using public water supply service area polygons where available, and Census place names and borders where service area polygons were unavailable. They were then assigned an atrazine concentration based on the amount of atrazine estimated by the USGS models for that utility. Mothers who reported using well water were assigned an atrazine concentration using the same method as the groundwater intakes. Mothers with more than one residential address during the critical exposure period were assigned a weighted value based on the atrazine concentration and the number of weeks at each address. We excluded mothers without a full residential history, mothers using public water who were not successfully matched to a public water utility, and mothers using a utility that was not successfully assigned an atrazine concentration by one of the two USGS models. This reduced our sample size to 123 cases (35.9 % of the original sample) and 415 controls (29.2 % of original sample). We examined distributions across covariates for those excluded to help characterize any selection bias that might be introduced by these exclusions.

We then estimated the daily amount of atrazine consumed via drinking water by a mother by multiplying the estimated atrazine concentration in a mother’s raw water supply by the self-reported amount of water consumed daily by the mother. The self-reported number of glasses consumed ranged from 0 to 24 8-oz glasses of water daily. Because it is unlikely that pregnant women are consuming no water, we converted the women who reported drinking 0 glasses to missing and excluded from further analyses. We then multiplied the number of 8-ounce glasses by 0.237 to convert to liters and estimate a total consumption in micrograms.

The distributions of maternal socioeconomic, demographic, and behavioral characteristics and hypospadias cases and controls were examined. We used multivariable logistic regression analysis to estimate the odds ratio for a hypospadias birth using two exposure measures of interest: estimated concentration of atrazine in a mother’s raw water supply and estimated daily maternal atrazine consumption. We estimated crude and adjusted odds ratios for hypospadias for each exposure measure. We estimated odds ratios stratified by state, and then estimated an overall odds ratio using a random effects model with state as the group variable. Covariates used for adjustment were selected based on existing literature, and included private well use (yes, no), residential use of water filtration (yes, no), state of residence (North Carolina, Iowa, Arkansas, Texas), maternal age (14–19, 20–24, 25–29, 30–34, 35–39, and over 40), maternal race/ethnicity (non-Hispanic white, non-Hispanic black, Hispanic, and other), multiple or singleton birth, previous pregnancies (yes, no), maternal education (less than high school, high school, or more than high school), maternal diabetes (yes, no), maternal high blood pressure (yes, no), maternal body mass index (BMI) (less than 18.5, 18.5–25, 25–30, and over 30), choline use (less than 187.4 mg, 187.4–249.6 mg, 249.6–336.4 mg, and greater than 336.4 mg, consistent with Carmichael et al. [14]), and use of artificial reproductive technology (yes, no). Analyses were performed using Stata version 13.1 (StataCorp LP, College Station, TX, USA).

## Results and Discussion

Distributions of demographic characteristics (state of residence, age, multiple or singleton birth, previous pregnancy, and education), behavioral characteristics (use of private wells, water filtration, choline, and artificial reproductive technology), and health characteristics (BMI, diabetes, and high blood pressure), for mothers of hypospadias cases (*n =* 123) and mothers of male controls (*n =* 415) are presented in Table 1. Mothers of cases were more likely to be non-Hispanic white, more highly educated, and to have used fertility medications or procedures, and infants with hypospadias were more likely to be a result of a first pregnancy or a multiple birth. Mothers of cases were slightly more likely to report drinking 5 or more glasses of water a day. While controls were fairly evenly distributed amongst the 4 states, 79.6 % of cases lived in Arkansas and North Carolina, which is likely to be due to enrollment processes for NBDPS [13]. No significant differences in distributions were observed for reported use of a private well, residential use of water filtration, maternal age, maternal BMI, maternal diabetes, maternal high blood pressure, or maternal choline intake.

**Table 1:** Characteristics of NBDPS hypospadias cases and controls with estimated atrazine exposure, 1998-2005

	Cases			Controls		
Characteristic	*N*	%	Missing	*N*	%	Missing
*Demographic characteristics*						
State of residence **			0			0
Arkansas	49	39.8		85	20.5	
Iowa	17	13.8		103	24.8	
Texas	8	6.5		101	24.3	
North Carolina	49	39.8		126	30.4	
Maternal age			0			0
<20	10	8.1		43	10.4	
20-24	27	22.0		89	21.5	
25-29	25	20.3		118	28.4	
30-34	42	34.2		105	25.3	
≥35	19	15.5		60	14.5	
Maternal race/ethnicity**			0			0
Non-Hispanic white	94	76.4		242	58.3	
Non-Hispanic black	16	13.0		32	7.7	
Hispanic	8	6.5		106	25.5	
Other	5	4.1		35	8.4	
Maternal education**			0			0
<High school	5	4.1		86	20.7	
High school	30	24.4		111	26.8	
>High school	88	71.5		218	52.5	
Previous pregnancies**			0			0
No	55	44.7		122	29.4	
Yes	68	55.3		293	70.6	
Plurality*			0			0
Singleton birth	114	92.7		405	97.6	
Multiple birth	9	7.3		10	2.4	
*Behavioral characteristics*						
Private well use			2			9
No	85	70.3		292	71.9	
Yes	36	29.7		114	28.1	
Reported water consumption^+^			0			0
0 glasses	3	2.4		22	5.3	
1-4 glasses	77	62.6		217	52.3	
5 or more glasses	43	35.0		176	42.4	
Residential filtered tap water			2			9
No	84	69.4		304	74.9	
Yes	37	30.6		102	25.1	
Maternal choline intake^a^			0			0
<187.4 mg	27	22.0		72	17.4	
187.4 – 249.6 mg	30	24.4		85	20.5	
249.7 – 336.3 mg	34	27.6		104	25.1	
>336.4 mg	32	26.0		154	37.1	
Fertility medications or procedures*			0			4
No	111	90.2		393	95.6	
Yes	12	9.8		18	4.4	
*Health characteristics*						
Diabetes			0			1
No	115	93.5		374	90.3	
Yes	8	6.5		40	9.7	
High blood pressure			0			0
No	95	77.2		344	92.9	
Yes	28	22.8		71	17.1	
Maternal BMI			1			22
<18.5	6	4.9		20	5.1	
18.5-25	59	48.4		210	53.4	
25-30	25	20.5		84	21.4	
>30	32	26.2		79	20.1	

^+^ *p* < 0.1

*p < 0.05

**p < 0.01

^a^Categories for maternal choline intake from Carmichael et al. [14]

Raw distributions for estimated concentrations of atrazine for a mother’s water supply and for estimated daily atrazine consumption are presented in Table 2. When combining the data across all 4 states, mean and median concentrations were higher for controls than for cases for both estimated atrazine in water supply and estimated atrazine consumption. In addition, the mean was greater than the median for all estimates. We observed differences in this trend when the data were stratified by state. Specifically, this skewness may be due in part by the small number of cases in Iowa and Texas, which also had the highest concentrations of atrazine. Mean and median concentrations were similar for both cases and controls in North Carolina and Arkansas. The difference among states was greater for the estimated atrazine consumption because a small number of mothers consumed a large amount of water. In all states, the mean and median estimated atrazine in water supply was well below the EPA’s maximum contaminant level of 3 μg/L.

**Table 2:** Distribution of estimated atrazine in water supply and estimated atrazine consumption

	Cases				Controls			
	Mean	Median	IQR	Min, Max	Mean	Median	IQR	Min, Max
Estimated atrazine in water supply in AR, IA, TX, and NC (μg/L)	0.09	0.02	0.001–0.04	0.0001, 2.0	0.17	0.02	0.002–0.05	0.0001, 4.0
*Arkansas*	0.03	0.02	0.0004–0.03	0.00009, 0.31	0.02	0.02	0.005–0.03	0.0001, 0.31
*Iowa*	0.28	0.05	0.001–0.53	0.001, 0.95	0.47	0.45	0.002–0.66	0.0004, 4.02
*North Carolina*	0.02	0.02	0.0006–0.04	0.0002, 0.07	0.03	0.03	0.001–0.04	0.0002, 0.06
*Texas*	0.50	0.004	0.0004–0.99	0.0001, 1.98	0.17	0.005	0.001–0.13	0.0001, 3.94
Estimated atrazine consumption in AR, IA, TX, and NC (μg/day)	0.12	0.02	0.001–0.04	0.00004–3.75	0.14	0.02	0.002–0.06	0.00007–4.66
*Arkansas*	0.03	0.02	0.01–0.03	0.00004–0.22	0.02	0.01	0.003–0.03	0.00009–0.22
*Iowa*	0.29	0.06	0.001–0.47	0.0006–1.35	0.36	0.08	0.002–0.54	0.0002–2.86
*North Carolina*	0.02	0.01	0.0005–0.03	0.00008–0.08	0.03	0.02	0.001–0.05	0.0001–0.16
*Texas*	1.00	0.002	0.0004–3.28	0.0001–3.75	0.18	0.01	0.002–0.09	0.00007–4.66

Table 3 presents odds ratios for the interquartile range of exposure, reporting the increase or decrease in the odds of having a baby with hypospadias associated with an increase in atrazine concentration equal to the difference of the 75th and 25th percentiles of estimated exposure. For Arkansas and Texas, the unadjusted and adjusted odds ratios for the interquartile range of estimated concentration of atrazine in a mother’s water supply and a mother’s estimated daily atrazine consumption were elevated. Odds ratios for Iowa and North Carolina were below 1.0, but statewide estimates were relatively imprecise as evidenced by the wide confidence intervals (Table 3).

**Table 3:** Association between atrazine and hypospadias in the National Birth Defects Prevention Study, 1998–2005

	N (cases)	IQR	Crude OR	Adjusted OR^b^
*State level models for estimated atrazine in water supply*				
*Arkansas*	134 (49)	0.02	1.05 (0.88, 1.26)	1.02 (0.80, 1.24)
*Iowa*	120 (17)	0.63	0.64 (0.28, 1.42)	0.66 (0.26, 1.67)
*North Carolina*	175 (49)	0.003	0.97 (0.92–1.02)	0.97 (0.88, 1.08)
*Texas*	109 (8)	0.13	1.09 (0.97, 1.22)	1.22 (1.01, 1.48)
*State level models for estimated atrazine consumption*				
*Arkansas*	131 (49)	0.03	1.06 (0.83, 1.35)	1.40 (0.34, 5.78)
*Iowa*	106 (16)	0.54	0.85 (0.46, 1.57)	0.46 (0.02, 11.9)
*North Carolina*	171 (48)	0.05	0.50 (0.27, 0.91)	0.02 (0.00, 1.24)
*Texas*	105 (7)	0.09	1.07 (1.01, 1.12)	1.93 (1.02, 3.23)
*Estimated atrazine in water supply across states* ^*a*^	538 (123)	0.04	0.97 (0.94, 1.00)	1. 00 (0.97, 1.03)
*Estimated atrazine consumption across states* ^*a*^	513 (120)	0.05	0.99 (0.96 1.02)	1.02 (0.99, 1.05)

^a^All ORs reported for interquartile range, or an increase from the in atrazine concentration equal to the difference of the 75th and 25th percentiles

^b^ORs for random effects models using state as the group variable. Random effects models and models for Arkansas, Iowa, and North Carolina adjusted by private well use, residential use of filtered water, maternal age, maternal race/ethnicity, plurality, parity, maternal education, choline use, use of artificial reproductive technology, maternal diabetes, maternal high blood pressure, and maternal BMI. Models for Texas adjusted by only private well use, maternal age, maternal race/ethnicity, parity, maternal education, choline use, and maternal high blood pressure because of the small number of cases

The crude odds ratios for estimated atrazine in water supply and atrazine consumption, without stratification by state, were close to 1.0. After adjustment for multiple covariates the odds ratios for consumption were slightly increased above the null for the interquartile range of exposure.

Sensitivity analyses compared characteristics of women who were successfully assigned an atrazine exposure and women who were not successfully assigned an atrazine exposure. These analyses suggest that mothers who were excluded from the USGS metric were less likely to be mothers of hypospadias cases, less likely to use untreated water from private wells, more likely to live in Arkansas, more likely to be under age 30, and more likely to have a BMI under 18.5 or greater than 25. The mothers excluded from the metric were also more likely to identify as non-Hispanic white or non-Hispanic black. See Additional file 1 for additional information.

## Conclusions

After adjusting for maternal socioeconomic, demographic, and behavioral characteristics, we observed a weak association between hypospadias and maternal consumption of atrazine via drinking water during gestational weeks 6–16 in overall models. This was a slightly stronger association than that found by Meyer et al. [11], but a much weaker association than that found by Agopian et al. [12]. No association was observed in crude odds ratios, or when not accounting for the total amount of drinking water consumed. In state-level models, positive associations were observed in Arkansas and Texas, while the opposite trend was observed in North Carolina and Iowa.

Certain limitations should be considered when interpreting these results. While the USGS models that we employed allowed us to estimate atrazine concentrations in raw water supplies, they do not account for treatment at public water supplies. Water treatment practices may vary geographically, atrazine concentrations may vary seasonally, and the USGS models do not capture this geographic or temporal variation. In addition, other contaminants which we were unable to measure or estimate, particularly agricultural byproducts, may be correlated with atrazine in water supplies.

We did consider other exposure estimation techniques, first using monitoring data from the US Environmental Protection Agency, and then using the amount of atrazine applied at the county level. Both of these alternatives proved problematic. Monitoring data were not available for atrazine concentrations below the US Environmental Protection Agency’s Maximum Contaminant Level, which prevented us from considering associations between hypospadias and lower levels of atrazine. Further, the total amount of atrazine applied at the county level would not have allowed us to consider the interaction between atrazine concentrations in water supplies and maternal water consumption. The USGS water models allowed us to estimate atrazine concentrations for private wells, which are not regulated by the EPA, and for individual water supplies with atrazine concentrations below the EPA’s Maximum Contaminant Level.

We cannot be sure that our models accurately predict maternal exposure to atrazine without a validated, repeated measure of atrazine in maternal urine during pregnancy, and the lack of more reliable data on atrazine exposure undoubtedly led to some misclassification when assigning maternal exposure status. Our exposure estimates also relied on self-reported water consumption, which may be prone to recall bias. Assuming that atrazine concentration misclassification and recall bias was largely random between case and control mothers, the results would have tended to be biased toward the null, although this does not guarantee that our estimate is an underestimate [23]. While our exposure estimates therefore should not be used in a quantitative risk assessment, the continuous nature of our estimated exposure may be less prone to misclassification than a binary exposure variable and useful for hypothesis generation.

Another limitation was our inability to assign atrazine concentrations to many of the NBDPS women. Sensitivity analyses revealed that women who were not successfully assigned an atrazine concentration were more likely to live in Arkansas, which was associated with increased risk in this study. Women who were not successfully assigned an exposure were also more likely to be non-Hispanic white or non-Hispanic black, which were characteristics associated with decreased hypospadias risk in this study. They were also less likely to use private wells, more likely to be under age 30, and more likely to have a BMI under 18.5 or greater than 25, which were characteristics that were not associated with hypospadias risk in this study. In addition, a number of women were identified by state centers as eligible cases or controls, but were not successfully interviewed. It is therefore unclear how exclusion of these women may have influenced our results. Finally, some of the odds ratio estimates were based on smaller sample sizes and were imprecise.

This study also had several strengths. While other studies have looked at proximity to pesticide application, our modeled exposure estimates allowed us to consider exposure via drinking water as a potential mechanism for a possible association between atrazine and hypospadias. It also took advantage of the unique water consumption and other covariate data available through the National Birth Defects Prevention Study (NBDPS), which allowed us to improve our exposure assessment and control for confounding.

A further strength of this study was that all of the hypospadias cases were ascertained by population-based birth defect surveillance systems, and underwent a detailed clinical review and classification prior to inclusion in the study. Cases with known genetic or chromosomal abnormalities were excluded. This resulted in a more etiologically and pathogenically homogenous case group.

Our models of maternal consumption of atrazine via drinking water (OR 1.02 (95 % CI 0.99-1.05)) may provide limited support for the hypothesis that atrazine may be associated with male genitourinary malformations in humans, although we could not exclude the possibility of a null association, given the limitations described above. Our results are not intended to be used in lieu of an exposure risk assessment, but rather to generate hypotheses about the trends and patterns of associations between atrazine and hypospadias. Further research including a larger sample size and better exposure characterization would be useful to provide a more definitive characterization of the potential effects of atrazine.

## Abbreviations

BMI, body mass index; CHEEC, Center for Health Effects of Environmental Contamination; NBDPS, National Birth Defects Prevention Study; USGS, United States Geological Survey; WARP, Watershed Regressions for Pesticides

## Additional file

Additional file 1: Characteristics of women successfully assigned an atrazine exposure and women who were not successfully assigned an atrazine exposure. (DOCX 19 kb)
