# Supplementary material for: Hypospadias and maternal exposure to atrazine via drinking water in the National Birth Defects Prevention study
Source: Environ Health. 2016 Jul 15;15:76. doi: 10.1186/s12940-016-0161-9 (PMC4946150; doi:10.1186/s12940-016-0161-9)
Supplement: Additional file 1: — Characteristics of women successfully assigned an atrazine exposure and women who were not successfully assigned an atrazine exposure. (DOCX 19 kb) [file 12940_2016_161_MOESM1_ESM.docx]

*Supplementary materials: Characteristics of women successfully assigned an atrazine exposure and women who were not successfully assigned an atrazine exposure.*

|  | **Included in USGS metric (N = 513)** | **Excluded from USGS metric (N = 1,252)** | **P-value** |
| --- | --- | --- | --- |
| Hypospadias |  |  | <0.01 |
| Controls | 393 (76.6%) | 1,029 (82.2%) |  |
| Cases | 120 (23.4%) | 223 (17.8%) |  |
| Private well use |  |  | <0.01 |
| No | 367 (71.5%) | 993 (98.5%) |  |
| Yes | 146 (28.5%) | 15 (1.5%) |  |
| State of Residence |  |  | <0.01 |
| Arkansas | 131 (25.5%) | 459 (36.7%) |  |
| Iowa | 106 (20.7%) | 353 (28.2%) |  |
| Texas | 105 (20.5%) | 324 (25.9%) |  |
| North Carolina | 171 (33.3%) | 116 (9.3%) |  |
| Maternal age |  |  | <0.01 |
| <20 | 50 (9.8%) | 203 (16.2%) |  |
| 20-24 | 112 (21.8%) | 313 (25.0%) |  |
| 25-29 | 134 (26.1%) | 370 (29.6%) |  |
| 30-34 | 139 (27.1%) | 252 (20.1%) |  |
| ≥35 | 78 (15.2%) | 114 (9.1%) |  |
| Maternal race/ethnicity |  |  | <0.01 |
| Non-Hispanic white | 318 (62.0%) | 810 (64.8%) |  |
| Non-Hispanic black | 47 (9.2%) | 138 (11.0%) |  |
| Hispanic | 110 (21.4%) | 262 (20.9%) |  |
| Other race/ethnicity | 38 (7.4%) | 41 (3.3%) |  |
| Maternal education |  |  | 0.17 |
| Less than high school | 87 (17.0%) | 215 (17.8%) |  |
| High school | 135 (26.3%) | 364 (30.2%) |  |
| More than high school | 291 (56.7%) | 627 (52.0%) |  |
| Previous pregnancies |  |  | 0.50 |
| No | 167 (32.6%) | 426 (34.3%) |  |
| Yes | 349 (67.4%) | 826 (65.7%) |  |
| Plural births |  |  | 0.87 |
| Singleton | 495 (96.5%) | 1,210 (96.7%) |  |
| Multiple | 18 (3.5%) | 42 (3.4%) |  |
| Filtered water |  |  | 0.06 |
| No | 376 (73.3%) | 792 (77.7%) |  |
| Yes | 137 (26.7%) | 227 (22.3%) |  |
| Diabetes |  |  | 0.97 |
| No | 468 (91.2%) | 1136 (91.2%) |  |
| Yes | 45 (8.8%) | 110 (8.8%) |  |
| High blood pressure |  |  | 0.12 |
| No | 419 (81.7%) | 1056 (84.7%) |  |
| Yes | 94 (18.3%) | 191 (15.3%) |  |
| Maternal BMI |  |  | 0.05 |
| <18.5 | 22 (4.5%) | 87 (7.3%) |  |
| 18.5-25 | 256 (52.2%) | 587 (49.2%) |  |
| 25-30 | 108 (22.0%) | 301 (25.3%) |  |
| >30 | 104 (21.2%) | 217 (18.2%) |  |
| Fertility medications or procedures |  |  | 0.16 |
| No | 484 (94.4%) | 1,150 (95.9) |  |
| Yes | 29 (5.7%) | 49 (4.1%) |  |
| Maternal choline intake |  |  | 0.84 |
|  | 97 (18.9%) | 225 (18.3%) |  |
|  | 105 (20.5%) | 262 (21.3%) |  |
|  | 130 (25.3%) | 331 (26.9%) |  |
|  | 181 (35.3%) | 413 (33.6%) |  |
